# The duo of precision imaging: computed tomography and optical coherence tomography give new opportunities for the diagnosis and conservative treatment of coronary plaque erosion—a case report

**DOI:** 10.1093/ehjcr/ytaf099

**Published:** 2025-02-27

**Authors:** Denitsa Meteva, Elaaha Anwari, Ulf Landmesser, Youssef S Abdelwahed

**Affiliations:** Department of Cardiology, German Heart Center of the Charite, Hindenburgdamm 30, Berlin 12203, Germany; Department of Cardiology, German Heart Center of the Charite, Hindenburgdamm 30, Berlin 12203, Germany; Department of Cardiology, German Heart Center of the Charite, Hindenburgdamm 30, Berlin 12203, Germany; Department of Cardiology, German Heart Center of the Charite, Hindenburgdamm 30, Berlin 12203, Germany

A 36-year-old male with chest pain after physical exercise presented in our emergency department without signs of ischaemia in the electrocardiogram (ECG). Besides regularly smoking e-cigarettes no other cardiovascular risk factors were noted. However, the initial high-sensitive troponin T test was positive at 19 ng/L [upper reference limit (URL)< 14 ng/L] with a relevant 1-h-dynamic increase, indicating a non ST-elevation acute coronary syndrome (NSTE-ACS). Considering the low negative predictive value for cardiovascular disease, the patient underwent coronary computed tomography (CT) and CT pulmonary angiography. Pulmonary embolism was excluded, but coronary CT identified a hazy lesion in the mid left anterior descending (LAD) artery, indicative of either a low-attenuated plaque or potentially a thrombus formation (*Panels A* and *B*). While the VERDICT-Trial depicted coronary CT angiography as a highly sensitive [96.5% (confidence interval 94.9–97.8)] non-invasive imaging tool for the detection of culprit plaques in NSTE-ACS, differentiating between low-attenuated plaques and thrombus can still be challenging due to similar Hounsfield units. The engagement of photon-counting CT provides higher spatial resolution, leading to improved tissue characterization; however, its widespread clinical availability is currently limited to larger centres. Additionally, multi-phase acquisitions can aid in distinguishing between thrombus and plaque, as thrombi tend to retain contrast longer and plaques do not. Optimizing contrast timing protocols can further enhance differentiation. Anyways, subsequent coronary angiography with intravascular imaging is therefore often inevitable. In the current case, coronary angiography confirmed a mid-LAD thrombus (*Panel C*), while optical coherence tomography demonstrated a healthy proximal and distal vessel. The light-breaking properties of the red thrombus obscured the underlying plaque, suggesting probable erosion (*Panel D*). Without significant stenosis and with adequate flow area, no stent was implanted and patient received GPIIb/IIIa inhibitors for 24 h, followed by aspirin and ticagrelor for 12 months. The patient was discharged symptom-free with normal left ventricular ejection fraction. A second coronary angiography with optical coherence tomography (OCT) after 2 months revealed no residual thrombus and a clear lipid plaque without vulnerability signs like thin cap fibroatheroma or minimal lumen area (MLA) < 4 mm² (*Panels E* and *F*). This case demonstrates the rising value of non-invasive coronary CT imaging in the diagnosis and potentially management of NSTE-ACS, highlighting the need for improvement of the current diagnostic algorithms through further identification and validation of specific computed tomography angiography (CTA)-characteristics of plaque erosion beyond the currently known total plaque volume (TPV) ≤ 116 mm^3^ and high risk plaque (HRP) features ≤1. Further studies utilizing CTA and OCT are therefore essential, with AI-driven deep learning models already in development and showing promising results in the non-invasive diagnosis of plaque erosion. Leveraging AI to identify key plaque erosion features on coronary CT could enhance risk stratification, guide decision-making, and optimize invasive management by pre-selecting acute coronary syndrome (ACS) patients for whom non-invasive treatment may serve as an alternative to standard care.

**Figure:**
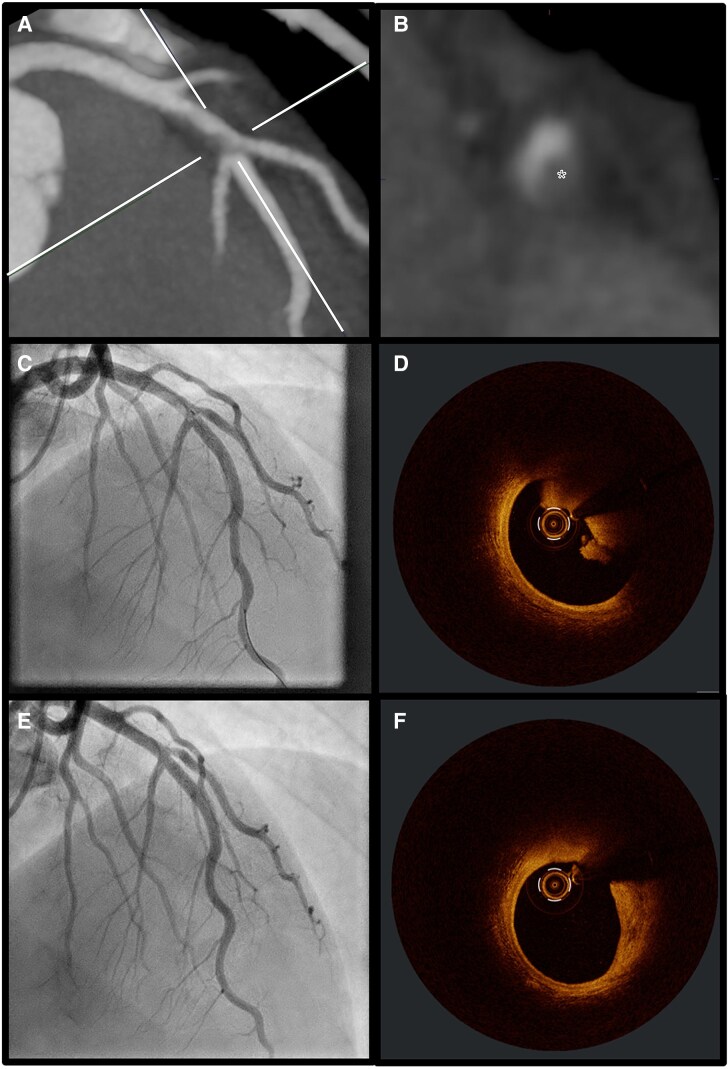


##  

**Consent:** The authors confirm that written consent for the submission and publication of this case report, including images and associated text, has been obtained from the patient in accordance with COPE guidance.

**Funding:** None declared.

## Data Availability

The data underlying this article will be shared on request to the corresponding author.

